# Effects of body size and load carriage on lower-extremity biomechanical responses in healthy women

**DOI:** 10.1186/s12891-021-04076-0

**Published:** 2021-02-24

**Authors:** Ginu Unnikrishnan, Chun Xu, Michael Baggaley, Junfei Tong, Sahil Kulkarni, W. Brent Edwards, Jaques Reifman

**Affiliations:** 1 Department of Defense Biotechnology High Performance Computing Software Applications Institute, Telemedicine and Advanced Technology Research Center, United States Army Medical Research and Development Command, FCMR-TT, 504 Scott Street, Ft. Detrick, MD 21702-5012 USA; 2 The Henry M. Jackson Foundation for the Advancement of Military Medicine, Inc., 6720A Rockledge Drive, Bethesda, MD 20817 USA; 3 Human Performance Laboratory, Faculty of Kinesiology, University of Calgary, Calgary, AB T2N 1N4 Canada; 4 The McCaig Institute for Bone and Joint Health, Cumming School of Medicine, University of Calgary, Calgary, AB T2N 1N4 Canada

**Keywords:** Joint kinematics and kinetics, Motion-capture data, Musculoskeletal finite-element analysis, Stress-fracture risk, Tibial strains

## Abstract

**Background:**

Musculoskeletal injuries, such as stress fractures, are the single most important medical impediment to military readiness in the U.S. Army. While multiple studies have established race- and sex-based risks associated with a stress fracture, the role of certain physical characteristics, such as body size, on stress-fracture risk is less conclusive.

**Methods:**

In this study, we investigated the effects of body size and load carriage on lower-extremity joint mechanics, tibial strain, and tibial stress-fracture risk in women. Using individualized musculoskeletal-finite-element-models of 21 women of short, medium, and tall statures (*n* = 7 in each group), we computed the joint mechanics and tibial strains while running on a treadmill at 3.0 m/s without and with a load of 11.3 or 22.7 kg. We also estimated the stress-fracture risk using a probabilistic model of bone damage, repair, and adaptation.

**Results:**

Under all load conditions, the peak plantarflexion moment for tall women was higher than those in short women (*p* < 0.05). However, regardless of the load condition, we did not observe differences in the strains and the stress-fracture risk between the stature groups. When compared to the no-load condition, a 22.7-kg load increased the peak hip extension and flexion moments for all stature groups (*p* < 0.05). However, when compared to the no-load condition, the 22.7-kg load increased the strains and the stress-fracture risk in short and medium women (*p* < 0.05), but not in tall women.

**Conclusion:**

These results show that women of different statures adjust their gait mechanisms differently when running with external load. This study can educate the development of new strategies to help reduce the risk of musculoskeletal injuries in women while running with external load.

## Background

Overuse musculoskeletal injuries, such as stress fracture, account for 70% of all injuries among military recruits and active Soldiers in the U.S. Army [1]. The high incidence of stress fracture in recruits is attributed to strenuous exercises and other physical activities performed during basic combat training [2], specifically, walking and running while carrying a heavy load. Multiple studies have shown that stress fracture is multifactorial in nature and is influenced by modifiable factors, such as training intensity, training duration, and load carriage, as well as non-modifiable factors, such as race, sex, and body size [3–5]. For example, increased duration and frequency of training sessions as well as heavy load carriage can increase the risk of stress fracture [5]. Similarly, there is overwhelming evidence to support the fact that Whites compared to Blacks, women compared to men, and older individuals compared to younger ones are more susceptible to stress fracture [6, 7]. However, the role of certain physical characteristics, such as body size, on stress-fracture risk is less conclusive. For example, while Kelly et al. reported that shorter and lighter individuals have a higher incidence of stress fracture [8], Knapik et al. [9] and Välimäki et al. [10] reported that men who are taller are at an increased risk of stress fracture. In contrast, other studies have shown no association between incidence of stress fracture and body size [7, 11].

Body size influences an individual’s capacity for physical performance [12, 13] and the biomechanics of locomotion [14, 15]. For example, with increasing body height, cross-sectional area of muscle and bone increases [12], which will also lead to higher force development capacity (i.e., muscle strength) [13]. Similarly, individuals with greater body mass tend to present an extended knee posture during walking in order to mitigate body size-related increase in the knee moment [14, 15]. In addition, walking or running with a load causes alteration in gait kinematics and kinetics [16]. Specifically, walking and running with a load reduces stride length, increases cadence, increases hip and knee flexion angles and moments [17, 18], and increases tibial mechanical stress, which could potentially elevate the risk of tibial stress fracture [19]. However, to date, it is still unclear how load carriage influences the biomechanics of running and the associated risk of stress fracture as a function of body size. Such an investigation is critical, especially because there are considerable differences in the body size of enlisted U.S. female Soldiers [height: 148.0–178.1 cm, mass: 46.4–98.3 kg [20]], and regardless of their body size, each Soldier is required to complete the same physical activities while carrying the same load.

Recently, using individualized musculoskeletal-finite-element (M/FE) models, we determined the effects of load carriage on joint mechanics (i.e., kinematics and kinetics) and the spatiotemporal distribution of tibial stress in three women of different sizes, walking with or without load [21]. In the individualized M/FE models, we accounted for subject-specific features, such as body size, bone morphology, and bone mineral density distribution, in addition to motion characteristics. When compared to the predictions from a generic musculoskeletal model, joint reaction (i.e., contact) forces (JRFs) predicted by the individualized musculoskeletal models differed by as much as 22% in the knee and 26% in the ankle [21]. Moreover, tibial stresses predicted by the individualized models were 31% greater than those predicted by the generic finite element (FE) models. These results highlight the importance of individualized M/FE models in assessing the mechanical loads acting on different individuals performing the same activity.

In the current study, we used individualized M/FE models to investigate the effects of body size and load carriage on the joint kinematics and kinetics, tibial strain, and the risk of stress fracture in women when they ran at 3.0 m/s (i.e., a 9.0-min/mile pace) on a treadmill, without and with a load of 11.3 or 22.7 kg. The objective of this study is to determine the effects of women body size in joint kinematics and kinetics as well as strain in the tibia as a function of load carriage during level running.

Using a probabilistic stress-fracture prediction model [22–24], we also investigated the risk of stress fracture in these women resulting from running 4.8 km/day (i.e., 3.0 miles/day) for 100 consecutive days [22]. We hypothesized that the changes in the joint mechanics, specifically, those related to peak joint angles, forces, and moments, as well as the strain in the tibia and the likelihood of stress fracture in women running with a load are dependent on body size.

## Methods

### Imaging and motion-capture data

As reported previously [19], based on sample-size calculations, we enrolled 21 young, healthy women between the ages of 18 to 21 years [mean = 19.6 and standard deviation (SD) = 0.9 years], with a body mass index between 19 and 25 kg/m^2^ [mean = 22.2 and SD = 1.9 kg/m^2^]. We measured the height of each subject using a wall-mounted stadiometer and their body mass using a calibrated electronic scale (Table 1). Body mass index was calculated as mass (kg) divided by the square of height (m^2^). We assigned each subject to one of three stature groups determined a priori: *short* (*n* = 7; height, 149–160 cm; mass, 48–61 kg), *medium* (*n* = 7; height, 161–166 cm; mass, 54–64 kg), or *tall* (*n* = 7; height, 172–177 cm; mass, 59–72 kg). The subjects in the short, medium, and tall groups are less than the 30th percentile, between the 45th and 65th percentile, and greater than the 90th percentile of the U.S. female Soldier population, respectively [20]. All subjects were free of self-reported bone disorders or lower-limb injuries 3 months prior to data collection. For each subject, we obtained quantitative computed tomography (CT; GE Discovery Scanner, General Electric Medical System, Milwaukee, WI) scans of the left leg with an in-plane resolution of 0.49 × 0.49 mm^2^ and a slice thickness of 0.63 mm (Acquisition setting: 120 kVp and 200 mAs). Each CT scan also included a calibration phantom with known calcium hydroxyapatite concentrations (QRM, Moehrendorf, Germany).

**Table 1:** Anthropometric characteristics of 21 young, healthy subjects (*n* = 7 in each group) considered in the study. Subjects ranged from 18 to 21 years of age, with a body mass index (BMI) between 19 and 25 kg/m^2^. The data are presented as mean ± one standard deviation

Group	Age (years)	Mass (kg)	Height (cm)	BMI (kg/m^2^)
Short	19.6 ± 1.0	54.2 ± 5.0	154.6 ± 3.7	22.7 ± 2.3
Medium	19.3 ± 0.8	60.5 ± 3.9	163.5 ± 1.9	22.7 ± 1.7
Tall	20.0 ± 1.2	64.9 ± 5.1	174.0 ± 1.9	21.4 ± 1.8

We also acquired motion-capture data using an eight-camera motion analysis system (Vicon Nexus, Centennial, CO) that tracked 42 retroreflective markers secured on the subject’s body, including anatomical landmarks and segmental-tracking markers. Specifically, we placed the markers on the left and right anterior and posterior iliac crest, greater trochanters, femoral condyles, heel, and metatarsals. We collected the motion-capture data (at a frequency of 200 Hz) and ground reaction forces (GRF, frequency of 1000 Hz) while the subjects ran at 3.0 m/s on an instrumented treadmill (Bertec, Columbus, OH), carrying no load (0 kg, baseline model) or a load of 11.3 or 22.7 kg (i.e., 25 or 50 lb) using an adjustable weight vest (V-max, Rexburg, ID) [19]. These selected loads are representative of those carried by U.S. Soldiers during training and field operations [25]. We calculated stride frequency by using the time between successive ipsilateral foot contacts, and stride length by dividing the speed of the treadmill by the stride frequency while accounting for differences between ipsilateral foot contact positions. We received approval for the study from the Human Research Protection Office at the U.S. Army Medical Research and Development Command (Ft. Detrick, MD) and the Conjoint Health Research Ethics Board of the University of Calgary (Calgary, AB, Canada). All procedures were performed in accordance with applicable Department of Defense, U.S. Army, and U.S. Army Medical Research and Development Command human subject protection requirements. We obtained informed written consent from each participant prior to the study.

### Individualized musculoskeletal analysis

Using individualized musculoskeletal models, we determined joint kinematics and kinetics for each subject while they ran without load, or with a load of 11.3 or 22.7 kg. We developed the individualized models by scaling a generic female musculoskeletal model available in the AnyBody Modeling System™ (AnyBody Technology, Aalborg, Denmark) [20, 21, 26] and then morphed the tibiae of the generic model with the subject-specific tibiae. To scale the generic model, we used the anthropometric measurements of the individual and an optimization scheme that minimized the errors between markers defined in the model and those tracked in the experiment [27]. The individualized musculoskeletal models consisted of seven rigid bodies in the lower extremity, including pelvis, thigh, shank, and foot, with 55 Hill-type muscles per leg. Moreover, in the model we represented the hip joints as spherical joints and the knee and ankle joints as revolute joints [21]. In the musculoskeletal analyses, we computed muscle activities and reaction forces over one gait cycle using an inverse dynamics approach by minimizing the sum of the cubed muscle activities. We defined muscle activity as the ratio of muscle force divided by the muscle strength. We performed the inverse dynamics analyses, using the most representative stride from among the multiple strides that were acquired during the motion-capture experiments [28], to compute joint kinematics and kinetics over one gait cycle for each of the 21 subjects under three different load conditions (i.e., 0, 11.3, and 22.7 kg).

### Individualized finite element analysis

**Fig. 1:**
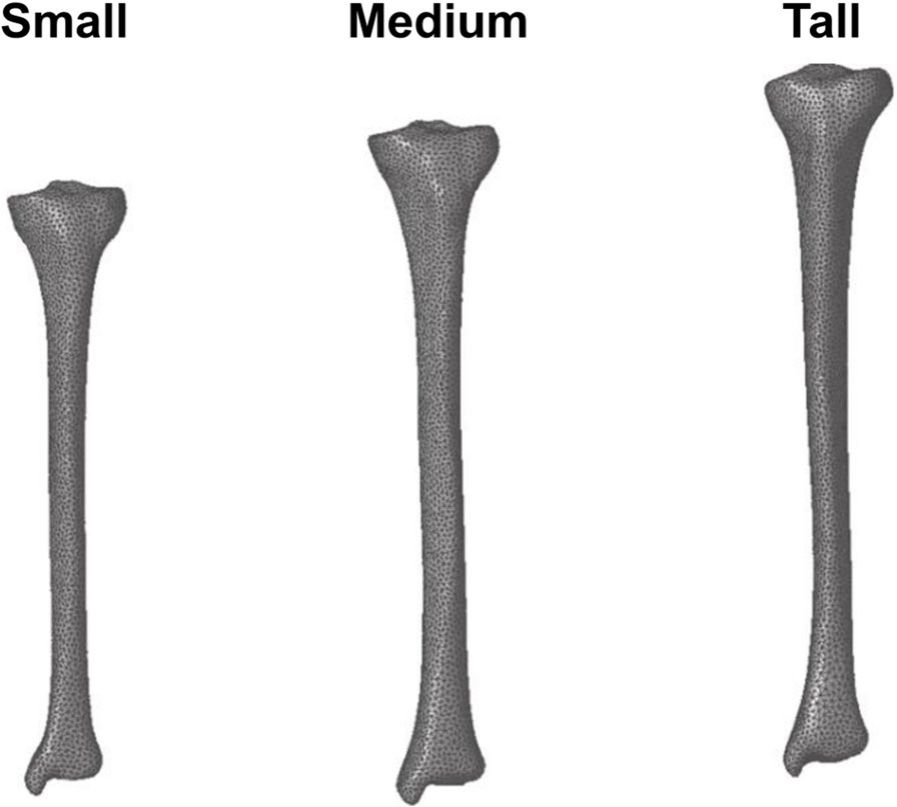
Representative finite element mesh of the left tibia for short-, medium-, and tall-statured women

Similar to our previous work [21], we developed subject-specific three-dimensional (3-D) FE models of the left tibia. We created a 3-D FE model for each of the 21 subjects using 3-Matic (Materialise, Leuven, Belgium) by generating 10-noded quadratic tetrahedral (C3D10) elements (average edge length varied from 3.0 to 3.5 mm) from the surface geometry of the subject-specific tibia, which was developed from the CT images (Fig. 1). We considered the bone (Poisson’s ratio of 0.325) and intramedullary tissue (Poisson’s ratio of 0.167) regions as linear elastic, isotropic materials. We calculated inhomogeneous material properties of the bone by converting the intensity of the CT images (defined in Hounsfield units) into apparent bone densities, and then to elastic moduli based on the intensity of the calibration phantom.

We specified the muscle forces as well as the bone forces and moments calculated from the individualized musculoskeletal analysis as loading conditions for the FE model. Specifically, we identified the muscle and ligament insertion points in the musculoskeletal model as FE constraint nodes, which we coupled to the outer surface of the tibial FE meshes. Through this procedure, on average, we specified 171 couplings between the muscle and the ligament nodes with the tibial FE meshes. We defined the bone-to-bone contact in a similar manner. Lastly, for each subject under each load condition, we performed a FE simulation (ABAQUS v6.17, Dassault Systèmes, Vélizy-Villacoublay, France) to determine the peak tibial strain for each element during one gait cycle. As the likelihood of stress fracture is dependent on the distribution of the peak strain in the bone [23, 24, 29], we also determined the 10th, 50th, and 90th percentile peak strains in the tibia during one gait cycle.

### Probabilistic model of stress-fracture likelihood

We determined the likelihood of stress fracture in the tibia using a probabilistic model [23, 24, 29], which considers bone failure due to fatigue damage and accounts for bone repair and adaptation. We represented the fatigue life of a bone F using an inverse power-law relationship given as F = C × ε^−6.6^, where C denotes a constant obtained from in vitro fatigue experiments on bone samples [29, 30] and ε represents the peak von Mises equivalent strain determined from the FE analyses. We represented bone adaptation, which increases the tibial areal moment of inertia and decreases the tibial strain, by assuming a constant lamellar bone deposition rate of 4 μm/day [31]. We accounted for changes in the tibial strain due to bone adaptation by calculating the equivalent tibial strain Δ ε_e_ for each element in the FE model, using the following relationship

1 $$ \Delta {\upvarepsilon}_{\mathrm{e}}={\left(\frac{1}{{\mathrm{t}}_d}\;{\int}_0^{{\mathrm{t}}_d}{\left(\mathrm{R}.\Delta \upvarepsilon \right)}^{\mathrm{n}}\mathrm{dt}\right)}^{1/\mathrm{n}} $$

where t_*d*_ denotes the total time in days over which adaptation takes place, R denotes the adaptation strain ratio, and n represents a material constant. In this study, we assumed n = 6.6, based on cortical bone fatigue experiments reported by Carter et al. [30]. We represented the cumulative probability of bone repair P_r_ as [23, 24, 29].

2 $$ {\mathrm{P}}_{\mathrm{r}}=1-\exp \left[-{\left(\frac{\mathrm{t}}{26}\right)}^2\right] $$

where t represents the time in days. To account for variability in the fatigue life F within a tibia, we divided the bone into eight regions (*N* = 8) based on similar strain values obtained from the FE analyses, and then determined the cumulative probability of failure of each region P_n_, with n = 1, 2, … ..., N, using the following relationship

3 $$ {\mathrm{P}}_{\mathrm{n}}=1-\exp \left[-\left(\frac{{\mathrm{V}}_{\mathrm{n}}}{{\mathrm{V}}_0}\right)\;{\left(\frac{\mathrm{t}}{{\mathrm{t}}_{\mathrm{f}}}\right)}^{1.2}\right] $$

where V_n_ denotes the volume of the nth elemental region, V_0_ denotes the volume of the bone sample used in fatigue experiments [22], and t_f_ denotes the reference time to fatigue failure (defined in days). The reference time is a function of the material strength of the bone, the equivalent strain ε_e_, and the number of loading cycles/day [23]. We represented the cumulative probability of fatigue failure of the entire tibia as

$$ {\mathrm{P}}_{\mathrm{f}}=1-{\prod}_{\mathrm{n}=1}^{\mathrm{N}}\left(1-{\mathrm{P}}_{\mathrm{n}}\right). $$

We combined the cumulative probabilities of repair P_r_ and fatigue failure P_f_ to determine the likelihood of stress fracture P_s_ in a tibia, which is given as

4 $$ {\mathrm{P}}_{\mathrm{s}}={\int}_0^{{\mathrm{t}}_d}{\mathrm{Q}}_{\mathrm{f}}\left(1-{\mathrm{P}}_{\mathrm{r}}\right)\kern0.28em \mathrm{dt} $$

where Q_f_ denotes the time differential of P_f_. We determined the risk of stress fracture for each subject by assuming that each subject ran 4.8 km/day (i.e., 3.0 miles/day) for 100 consecutive days [22]. We estimated the number of loading cycles/day by dividing the daily running distance by the subject’s stride length, which was measured during the motion-capture experiments.

### Statistical analysis

We determined the relationships between dependent variables and predictor variables using a linear mixed-effects analysis. We considered the outputs from the individualized musculoskeletal analysis (i.e., peak joint angles, moments, and forces) and those from the individualized FE analysis (i.e., tibial strains), as well as the likelihood of stress fracture as dependent variables. We considered load and stature group, which were categorical factors, as predictor variables. We included a random intercept for each subject to account for the within-subject dependence. To determine the statistical significance of a predictor variable, we used a likelihood-ratio test. We then performed a *post-hoc* Tukey test on the linear mixed-effects model for pairwise comparisons and corrected the results for multiple comparisons using the Holm-Bonferroni correction. We separately performed between-stature-group comparisons of the kinematic and kinetic responses, tibial strain, and the likelihood of stress fracture for each load condition, using analysis of variance. We represent all data as mean ± one SD, unless otherwise noted. We tested for statistical significance using a criterion of *p* < 0.05, and performed all analyses in the statistical software package R [32, 33].

## Results

### Joint kinematics and kinetics

**Fig. 2:**
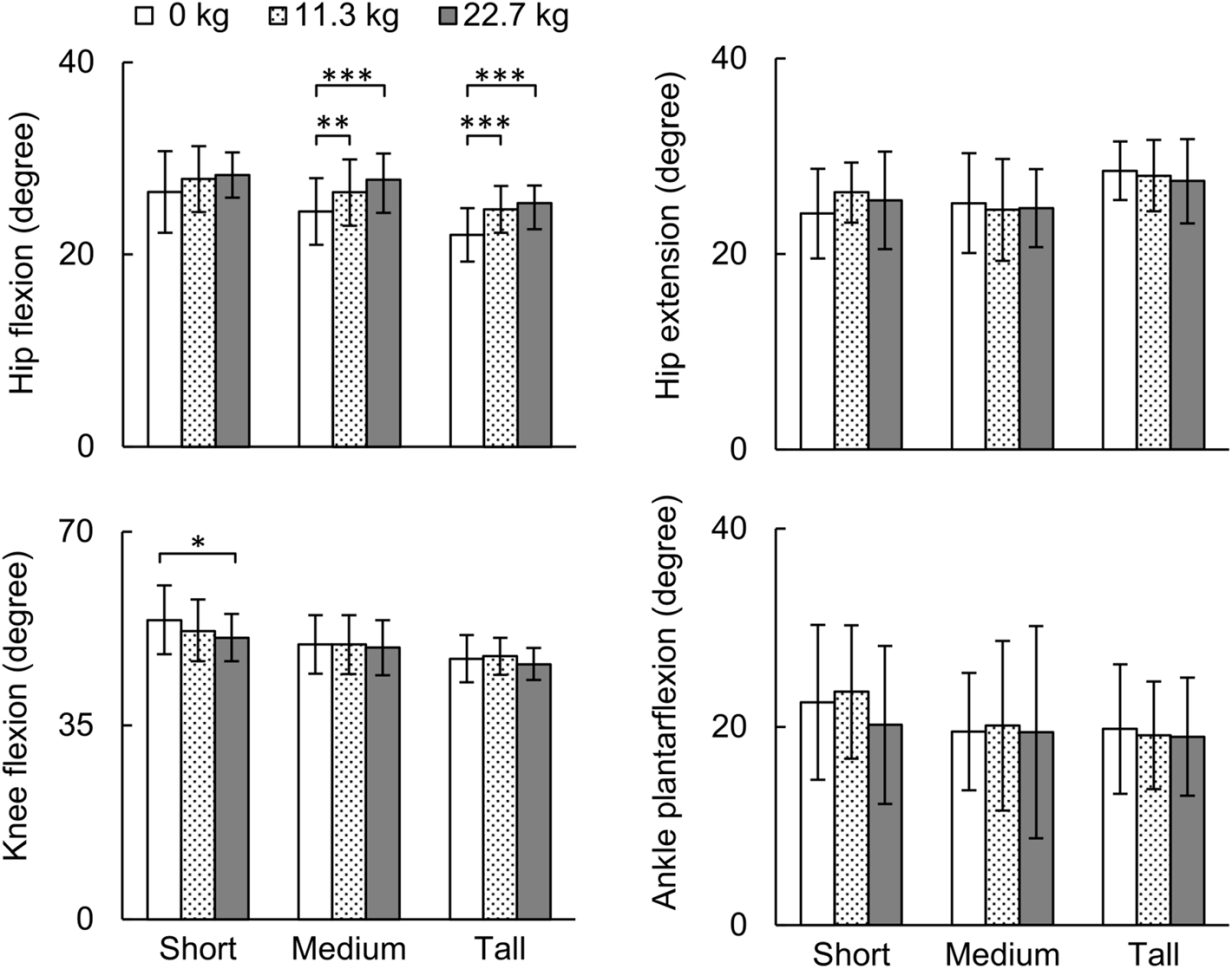
Comparison of peak joint angles at the hip, knee, and ankle under different loads during one gait cycle obtained from the individualized musculoskeletal analyses. Data are expressed as mean ± one standard deviation. *p < 0.05, **p < 0.01, ***p < 0.001

**Fig. 3:**
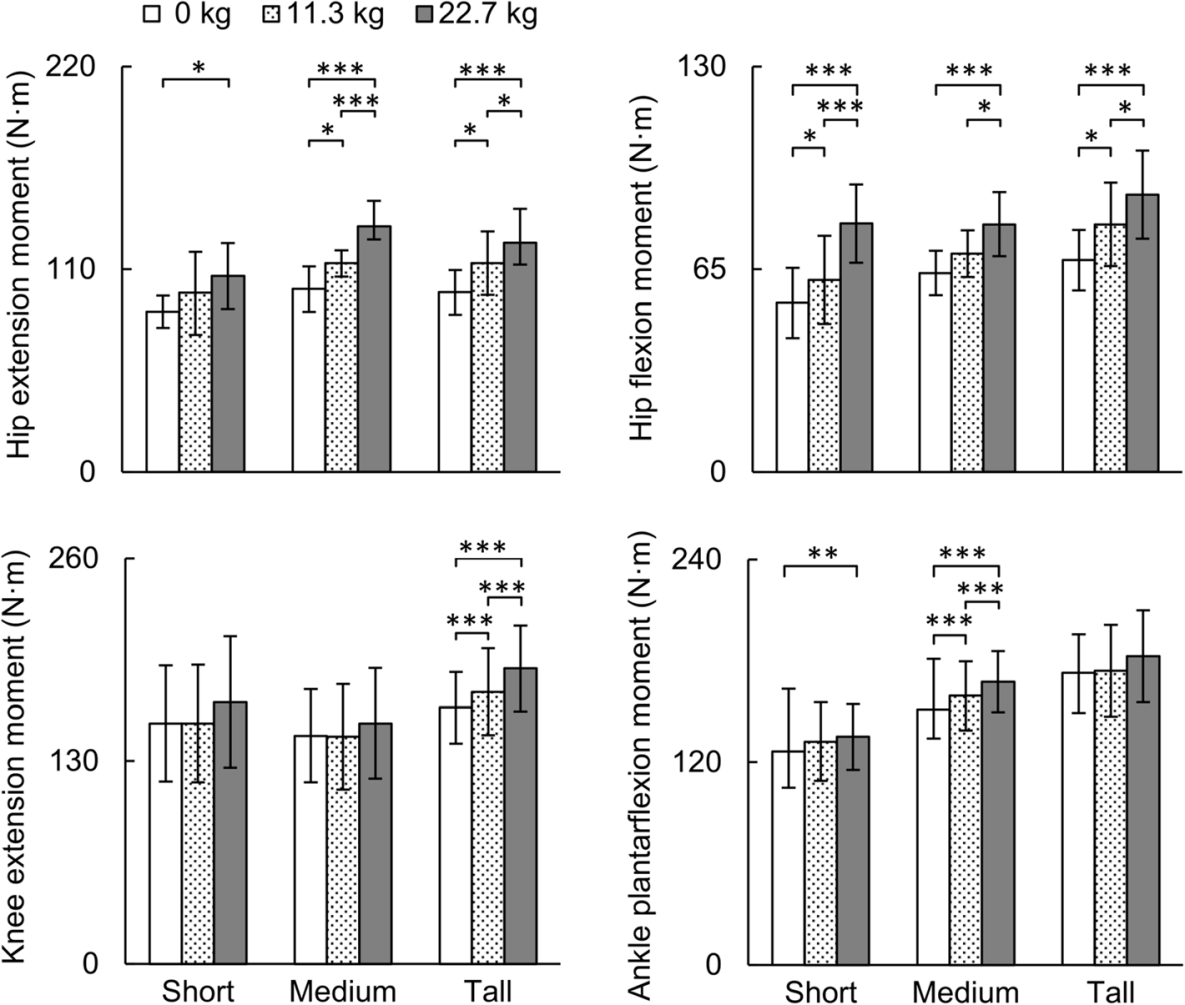
Comparison of peak joint moments at the hip, knee, and ankle under different loads during one gait cycle obtained from the individualized musculoskeletal analyses. Data are expressed as mean ± one standard deviation. *p < 0.05, **p < 0.01, ***p < 0.001

**Fig. 4:**
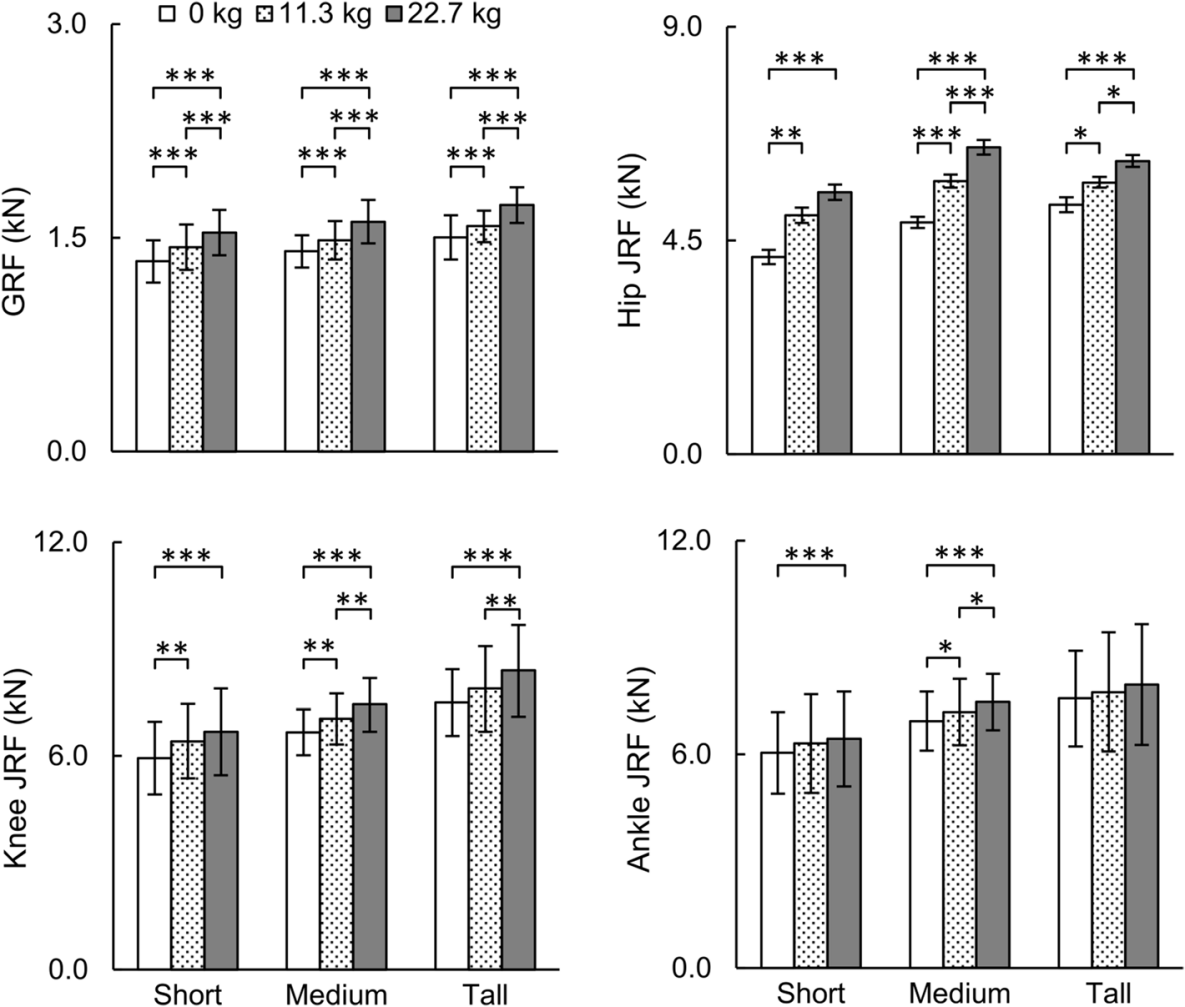
Comparison of peak ground reaction force (GRF) and peak joint reaction force (JRF) at the hip, knee, and ankle under different loads during one gait cycle obtained from the individualized musculoskeletal analyses. Data are expressed as mean ± one standard deviation. *p < 0.05, **p < 0.01, ***p < 0.001

Figures 2, 3 and 4 show the mean and SD of the peak kinematic and kinetic parameters predicted by the individualized musculoskeletal model. Under the baseline condition, the average peak hip flexion angle ranged from 22.0 degrees [(SD = 2.8), tall women] to 26.5 degrees [(SD = 4.2), short women] (Fig. 2, top left). In tall women, when compared to the baseline condition, a load of 11.3 or 22.7 kg significantly increased their peak hip flexion angle to 24.7 degrees [(SD = 2.4), an increase of 12.3%] or 25.4 degrees [(SD = 1.8), 15.5%], respectively (*p* < 0.001). Similarly, in the medium women group, we observed a statistically significant increase in the peak hip flexion angle when they ran with load (8.2% for 11.3 kg and 13.4% for 22.7 kg; *p* < 0.01). In short women, when compared to the baseline condition, a 22.7-kg load significantly reduced peak knee flexion angle during stance [i.e., from 54.1 (SD = 6.2) to 50.9 (SD = 4.3) degrees, a 5.9% reduction, *p* < 0.05]. We did not observe statistically significant differences in peak hip extension and ankle plantarflexion between the three different loads in short, medium, and tall women (Fig. 2, right).

When compared to the baseline condition, running with a load of 22.7 kg increased peak hip extension moment and flexion moment for the short, medium, and tall women (*p* < 0.05; Fig. 3). The peak knee extension moment increased from the baseline condition for tall women while running with a load of 11.3 or 22.7 kg (*p* < 0.001) but did not change for short and medium women. Running with a 22.7-kg load significantly increased the peak ankle plantarflexion moment in short women [i.e., from 126.3 (SD = 21.3) to 134.9 (SD = 19.3) N·m, *p* < 0.01] and medium women [i.e., from 151.3 (SD = 17.0) to 167.5 (SD = 18.0) N·m, *p* < 0.001], but not in tall women (Fig. 3).

As expected, the peak GRFs for the baseline condition were lower than the peak GRFs when running with a 11.3-kg or 22.7-kg load in short, medium, and tall women (*p* < 0.001; Fig. 4). Similarly, when running with a load, the peak JRF at the hip (*p* < 0.05) and the knee (*p* < 0.01) were higher than their corresponding values in the baseline condition. Moreover, running with a 22.7-kg load increased the average peak ankle JRFs by 6.7% for the short group and 8.7% for the medium group (*p* < 0.001).

We separately evaluated the effect of body size on joint kinematics and kinetics for each of the three load conditions. For the baseline condition, peak hip flexion moment and ankle plantarflexion moment for tall women were higher than the corresponding moments in short women by 25.9 and 36.8%, respectively (*p* < 0.05, Table 2). While running with an additional load of 11.3 or 22.7 kg, we observed differences in peak ankle plantarflexion moment (*p* < 0.01) and peak knee JRF (*p* < 0.05) between short and tall women. The peak hip and knee JRFs for tall women were higher than those in short women for the baseline conditions (*p* < 0.01).

**Table 2:** Peak joint angles, joint moments, and joint reaction forces at the hip, knee, and ankle when subjects ran without a load (0.0 kg), or with an additional load of 11.3 kg (25 lb) or 22.7 kg (50 lb), averaged within the group of short, medium, and tall women. The data are presented as mean ± one standard deviation

	0.0 kg				11.3 kg				22.7 kg			
	Short	Medium	Tall	*p*	Short	Medium	Tall	*p*	Short	Medium	Tall	*p*
**Stride frequency (strides/min)**												
	88.6 ± 5.6	87.3 ± 5.4	84.9 ± 3.4	0.39	90.5 ± 6.1	88.7 ± 5.1	85.9 ± 3.5	0.24	93.3 ± 6.1	90.1 ± 4.3	87.4 ± 3.2	0.09
**Ground reaction force (kN)**												
	1.3 ± 0.2	1.4 ± 0.1	1.5 ± 0.2	0.11	1.4 ± 0.2	1.5 ± 0.1	1.6 ± 0.1	0.16	1.5 ± 0.2	1.6 ± 0.2	1.7 ± 0.1	0.07
**Peak joint angle (degree)**												
Hip												
Flex	26.5 ± 4.2	24.5 ± 3.5	22.0 ± 2.8	0.09	27.9 ± 3.4	26.5 ± 3.5	24.7 ± 2.4	0.21	28.3 ± 2.4	27.8 ± 2.7	25.4 ± 1.8	0.06
Ext	24.2 ± 4.6	25.2 ± 5.1	28.5 ± 3.0	0.17	26.3 ± 3.1	24.5 ± 5.2	28.0 ± 3.7	0.30	25.5 ± 5.0	24.7 ± 4.0	27.5 ± 4.3	0.51
Knee												
Flex	54.1 ± 6.2	49.7 ± 5.3	47.1 ± 4.3	0.07	52.1 ± 5.5	49.7 ± 5.3	47.5 ± 3.3	0.23	50.9 ± 4.3	49.1 ± 5.0	46.1 ± 2.9	0.12
Ankle												
Plantar	22.5 ± 7.8	19.5 ± 5.9	19.8 ± 6.5	0.68	23.6 ± 6.7	20.1 ± 8.6	19.1 ± 5.4	0.48	20.2 ± 8.0	19.5 ± 10.8	19.0 ± 6.0	0.97
**Peak joint moment (N·m)**												
Hip												
Flex	54.3 ± 11.2^T*^	63.8 ± 7.1	68.0 ± 9.8^S*^	**0.04**	61.6 ± 14.1^T*^	70.1 ± 7.5	79.5 ± 13.4^S*^	**0.04**	79.7 ± 12.5	79.4 ± 10.3	89.1 ± 14.1	0.28
Ext	87.1 ± 8.6	99.4 ± 12.3	97.5 ± 12.2	0.09	97.1 ± 22.6	113.2 ± 7.1	113.4 ± 17.3	0.15	106.6 ± 17.8^M*^	133.5 ± 13.7^S*^	124.6 ± 18.3	**0.02**
Knee												
Ext	154.2 ± 37.2	146.1 ± 29.8	164.2 ± 23.0	0.51	154.1 ± 37.7	145.5 ± 33.7	174.4 ± 28.0	0.28	167.8 ± 42.1	154.1 ± 35.3	189.2 ± 27.4	0.21
Ankle												
Plantar	126.3 ± 21.3^T#^	151.3 ± 17.0	172.8 ± 23.8^S#^	**0.002**	132.2 ± 23.3^T*^	159.3 ± 20.3	174.2 ± 27.2^S*^	**0.01**	134.9 ± 19.3^M*,T†^	167.5 ± 18.0^S*^	182.7 ± 27.3^S†^	**0.002**
**Peak joint reaction force (kN)**												
Hip	4.2 ± 0.3^M#,T†^	4.9 ± 0.4^S#^	5.3 ± 0.5^S†^	**0.001**	5.0 ± 0.8	5.8 ± 0.6	5.7 ± 0.7	0.14	5.5 ± 0.5	6.5 ± 1.0	6.2 ± 0.6	0.07
Knee	5.9 ± 1.0^T*^	6.7 ± 0.6	7.5 ± 0.9^S*^	**0.01**	6.4 ± 1.0^T*^	7.0 ± 0.7	7.9 ± 1.2^S*^	**0.04**	6.7 ± 1.2^T*^	7.4 ± 0.8	8.4 ± 1.3^S*^	**0.03**
Ankle	6.0 ± 1.1	6.9 ± 0.8	7.6 ± 1.3	0.06	6.3 ± 1.4	7.2 ± 0.9	7.7 ± 1.7	0.17	6.4 ± 1.3	7.5 ± 0.8	8.0 ± 1.7	0.12

Bold p indicates statistically significant values obtained from between-stature-group comparison (using analysis of variance) for each of the three load conditions

*Flex* flexion *Ext* extension, *Plantar* plantarflexion

Superscripts *, ^#^, and ^†^ represent *p* < 0.05, *p* < 0.01, and *p* < 0.001, respectively, obtained from pairwise comparisons (using *post-hoc* Tukey test) between women of two particular stature groups (S-short, M-medium, T-tall), after performing the Holm-Bonferroni correction

### Tibial strains and stress-fracture risk

**Fig. 5:**
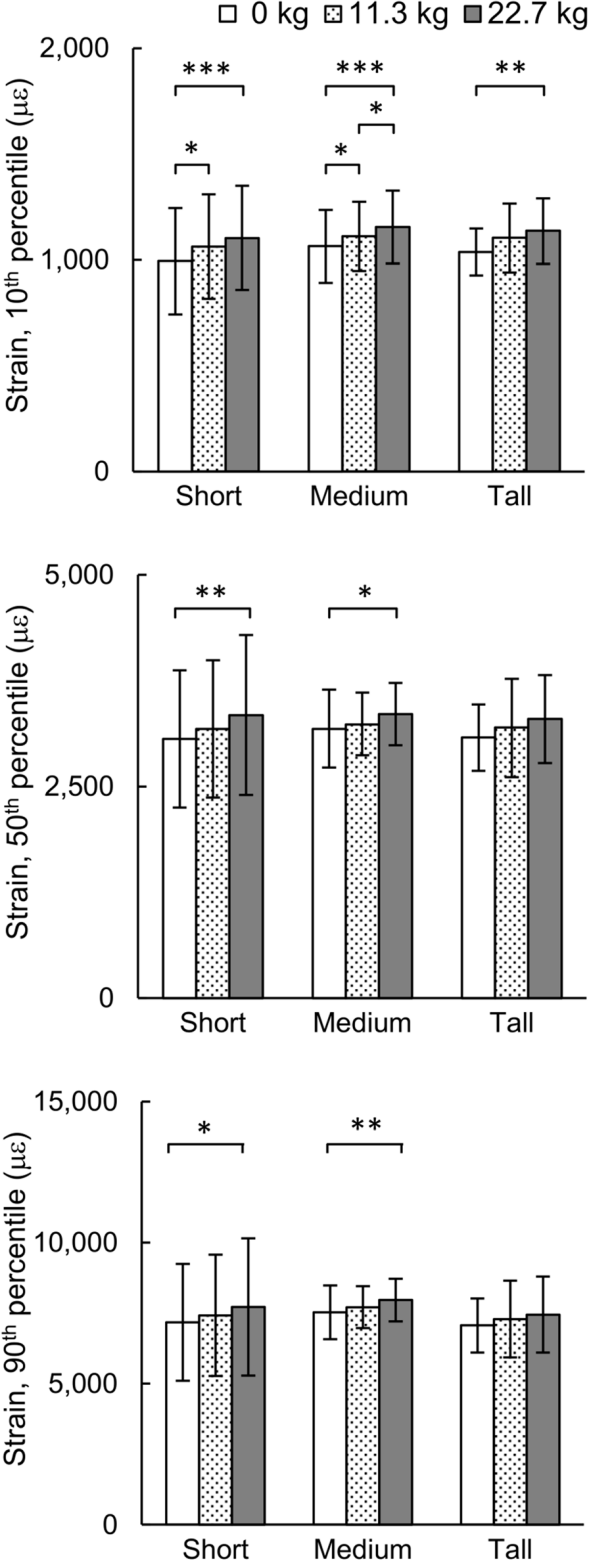
Comparison of von Mises strains (10th, 50th, and 90th percentile) under different loads for the short, medium, and tall groups during one gait cycle. Data are expressed as mean ± one standard deviation. *p < 0.05, **p < 0.01, ***p < 0.001

Figure 5 shows the predicted 10th, 50th, and 90th percentile peak tibial von Mises strain during the entire gait cycle in the tibial bone. The 10th percentile strain for the baseline condition was lower than the strain for the 11.3-kg and 22.7-kg load conditions for each of the three groups (*p* < 0.05). Compared to the baseline condition, an additional 22.7 kg significantly increased the 90th percentile von Mises strain in short women from 7183 (SD = 2069) to 7726 (SD = 2435) με (i.e., an increase of 7.6%) in short women and from 7531 (SD = 952) to 7968 (SD = 754) με (i.e., an increase of 5.8%) in medium women (*p* < 0.05; Fig. 5), but not in tall women. We did not observe any influence of body size on the tibial strain while running without or with a load of 11.3 and 22.7 kg.

**Fig. 6:**
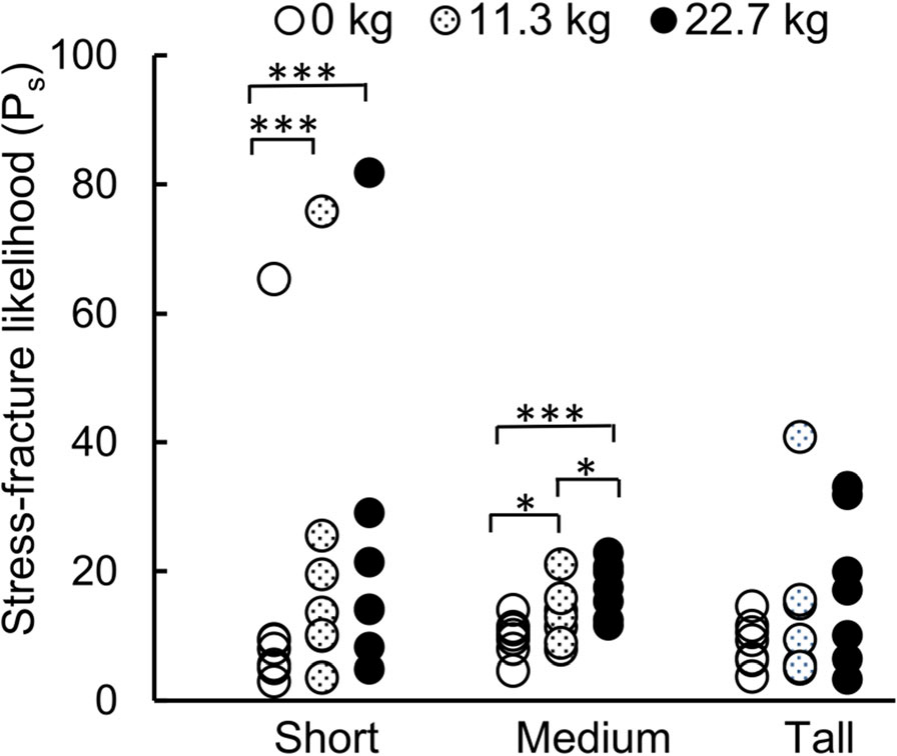
Stress-fracture likelihood P_s_ when subjects ran 4.8 km/day for 100 consecutive days under different loads for the short, medium, and tall groups (*n* = 7 in each group). *p < 0.05, ***p < 0.001

When compared to the baseline condition, an additional load of 22.7 kg increased the likelihood of stress fracture in short and medium women by 9.7 and 7.4%, respectively (*p* < 0.001; Fig. 6). However, similar to the effect of body size on the strains, we did not observe statistically significant differences in the likelihood of stress fracture between the short, medium, and tall groups in any of the three load conditions.

## Discussion

In this study, we developed individualized M/FE models for 21 women of *short* (*n* = 7), *medium* (*n* = 7), and *tall* (*n* = 7) statures and analyzed the effects of body size and load carriage on the kinematics and kinetics of lower extremity joints, tibial strain, and likelihood of stress fracture when these women ran without a load or with a load of 11.3 or 22.7 kg. When compared to the baseline (i.e., no-load) condition, we observed that the relative changes in the joint mechanics (i.e., kinematics and kinetics), tibial strain, and the risk of stress fracture when running with a load were dependent on body size (Figs. 2, 3, 4, 5 and 6). In partial agreement with our hypothesis, body size influenced the joint kinetics in women running with or without a load, but not the tibial strain and the likelihood of stress fracture.

Relative to the baseline condition, peak hip flexion angle increased with increasing load in medium and tall women (*p* < 0.01) but not in short women (Fig. 2). In addition, when compared to the baseline condition, peak hip flexion moment and extension moment of women running with a load of 22.7 kg increased for all stature groups (*p* < 0.05; Fig. 3). These results, which are in agreement with previous studies on women running with a load [21, 34], show engagement of hip muscles while performing a strenuous physical activity. Running with a load changed the kinematics and kinetics of the knee differently in the short, medium, and tall stature groups. For example, when compared to the baseline condition, running with a load of 22.7 kg reduced knee flexion angle during the stance phase in short women (*p* < 0.05), but not in tall women (Fig. 2). In contrast, with increasing load, the knee extension moment increased in tall women (*p* < 0.001) but not in short women (Fig. 3). When compared to the baseline condition, the reduction in the knee flexion angle in short women (Fig. 2) might reduce the ability of these women to dissipate forces while running with a 22.7-kg load. Increased knee stiffness, either due to a reduction in the knee flexion angle and/or increased knee flexion moment, is also considered as a risk factor for tibial stress fracture [35, 36].

Similar to the results of joint kinematics and kinetics, the change in the tibial strain (Fig. 5) and the likelihood of stress fracture with load carriage (Fig. 6), when compared to the baseline condition, was dependent on body size. For example, when compared to the baseline condition, running with 22.7-kg load increased the 50th and 90th percentile strain in the short and medium groups but not in the tall group. Similar to the change in the tibial strains, the likelihood of stress fracture in women resulting from running 4.8 km/day at 3.0 m/s for 100 consecutive days, increased with the load carried by women in the short and medium groups (*p* < 0.001). However, the differences in the strain and the likelihood of stress fracture for short, medium, and tall women under each of the three load conditions were not statistically significant, in spite of our model accounting for the important relationship between tibial size and stature [37]. It is interesting to note that, when compared to the other groups, the greater peak hip JRF for the baseline condition and peak knee JRF in the tall group for all load conditions (*p* < 0.05; Table 2) did not translate to higher strains or an increased risk of stress fracture for this group. We suspect that this observation could be due to stature-related differences in the geometry and material properties in the tibia, with the latter resulting from bone adaptation in response to mechanical forces generated during normal daily activity. These results also highlight the need for developing subject-specific M/FE models and combining the results of individuals with similar statures to perform group-level analyses.

The study has limitations. First, similar to our previous models [19, 21], we represented knee and ankle joints as revolute joints and did not include complex 3-D motions, including translation and rotation, of the joints. While inclusion of such complex motions can increase the fidelity of the model, revolute joints can adequately capture the kinematics and kinetics of the knee and ankle joints [38]. Therefore, we do not believe that incorporation of more complex 3-D motions in the musculoskeletal model would have changed the conclusions regarding the joint kinematics and kinetics as well as stress-fracture risk. Second, in the musculoskeletal model, we did not represent individualized muscle strength. Third, in the stress-fracture predictions, we did not consider any variation in gait mechanics due to muscle fatigue. Such an assumption may under-predict the likelihood of stress fracture, as fatigue may increase tibial strains and, thereby, the risk of stress fracture [39]. In addition, the probabilistic model assumes uniform bone remodeling and adaptation for all subjects. Although these assumptions are unlikely to affect the relative differences in the likelihood of stress fracture for the different load conditions for a subject, incorporation of muscle fatigue in the musculoskeletal model and an improved model of bone remodeling and adaptation [40] should be considered in the future to enhance the accuracy of stress-fracture predictions. Finally, we only quantified the effects of body size and load carriage for level running on a treadmill, without considering graded tests.

## Conclusion

In summary, using individualized M/FE model, we evaluated the effect of body size and load carriage on lower-extremity joint kinematics and kinetics, tibial biomechanics, and the likelihood of tibial stress fracture in 21 women running with or without a load. The results show that women of different statures adjust their gait mechanisms differently when running with a load. We also observed that in women running with or without a load, body size influences the joint kinetics, but not the prediction of tibial strains and the likelihood of stress fracture. These findings will help in quantifying the effect of load carriage on bone health and ultimately reduce the risk of musculoskeletal injuries of the lower extremities, such as knee injury and tibial stress fracture, in women.

## Data Availability

The FE-generated data and related analyses will be made available through a written request to the corresponding author, including a summary of the planned research.

## Abbreviations

3-D: Three-dimensional
BMI: Body mass index
CT: Computed tomography
GRF: Ground reaction force
JRF: Joint reaction force
M/FE: Musculoskeletal-finite-element
SD: Standard deviation
